# Drug Sensitivity of Vaccine-Derived Rubella Viruses and Quasispecies Evolution in Granulomatous Lesions of Two Ataxia-Telangiectasia Patients Treated with Nitazoxanide

**DOI:** 10.3390/pathogens11030338

**Published:** 2022-03-11

**Authors:** Raeesa Faisthalab, Suganthi Suppiah, Morna Dorsey, Kathleen E. Sullivan, Joseph Icenogle, Ludmila Perelygina

**Affiliations:** 1 Division of Viral Diseases, Centers for Disease Control and Prevention, Atlanta, GA 30333, USA; osp2@cdc.gov (R.F.); kpe9@cdc.gov (S.S.); jnmadice2@gmail.com (J.I.); 2 Department of Pediatrics, University of California San Francisco, San Francisco, CA 94143, USA; morna.dorsey@ucsf.edu; 3 Division of Allergy and Immunology, The Children’s Hospital of Philadelphia, Philadelphia, PA 19104, USA; sullivank@chop.edu

**Keywords:** immunodeficiency-related vaccine-derived rubella viruses (iVDRV), ataxia-telangiectasia, cutaneous granulomas, nitazoxanide, quasispecies

## Abstract

A strong association between rubella virus (RuV) and chronic granulomas, in individuals with inborn errors of immunity, has been recently established. Both the RA27/3 vaccine and wild-type RuV strains were highly sensitive to a broad-spectrum antiviral drug, nitazoxanide (NTZ), in vitro. However, NTZ treatment, used as a salvage therapy, resulted in little or no improvements of RuV-associated cutaneous granulomas in patients. Here, we report investigations of possible causes of treatment failures in two ataxia-telangiectasia patients. Although a reduction in RuV RNA in skin lesions was detected by real-time RT-PCR, live immunodeficiency-related vaccine-derived rubella viruses (iVDRV) were recovered from granulomas, before and after the treatments. Tizoxanide, an active NTZ metabolite, inhibited replications of all iVDRVs in cultured A549 cells, but the 50% and 90% inhibitory concentrations were 10–40 times higher than those for the RA27/3 strain. There were no substantial differences in iVDRV sensitivities, neither before nor after treatments. Analysis of quasispecies in the E1 gene, a suspected NTZ target, showed no effect of NTZ treatments on quasispecies’ complexity in lesions. Thus, failures of NTZ therapies were likely due to low sensitivities of iVDRVs to the drug, and not related to the emergence of resistance, following long-term NTZ treatments.

## 1. Introduction

Rubella virus (RuV, the family *Matonaviridae*, genus *Rubivirus*) is known to cause asymptomatic or mild infections that are quickly resolved in immunologically competent individuals [1,2]. RuV can establish persistent infections in fetuses and immune-privileged body sites, and cause substantial pathologies, such as congenital rubella syndrome (CRS), post-rubella encephalitis, arthritis, or Fuchs’ uveitis [3]. While successful vaccination campaigns, using live attenuated RuV vaccines have eliminated rubella and CRS in the Americas, and many countries worldwide, there are people with underlying conditions who are not recommended for vaccination, as they may exhibit adverse reactions [4,5]. One such population is patients with inborn errors of immunity (IEI). Around 1–4% of children with IEI have been observed to exhibit inflammatory granulomatous lesions, in multiple locations, including their skin and visceral organs [6]. 

Granulomas are formed by clusters of immune cells that surround and encapsulate a chronic antigenic trigger (pathogen or foreign body) but cannot eliminate it [7]. Granulomas often manifest as red-brown nodules with a plaque-like appearance [8]. There is strong evidence that the RuV vaccine can establish persistent infections in IEI patients and plays a role in granuloma development [9,10,11]. Infectious immunodeficiency-related vaccine-derived rubella viruses (iVDRVs) have been recovered from granuloma biopsies from IEI patients. The degree of evolutionary divergence in the iVDRVs has been directly correlated with the duration of iVDRV persistence in the patient, suggesting the continuous replication and sequence evolution of the RuV vaccine in IEI patients [12]. The iVDRVs persisted in granulomatous lesions, as a diverse population of closely related quasispecies [12].

No effective antiviral treatments for chronic rubella-associated diseases are currently available. We have previously demonstrated that the antiparasitic drug, nitazoxanide (NTZ), with broad spectrum antiviral capabilities, can effectively inhibit replication of RA27/3 vaccine virus and wild-type RuV of different genotypes in A459 cells, with an IC50 of 1 µM [13]. It is currently unknown whether NTZ can inhibit replication of iVDRV strains. NTZ was shown to be effective against a variety of viruses, but its antiviral mechanisms are not presently clear [14,15]. The drug appears to affect different stages of RuV replication cycle by inhibiting total RNA synthesis in the infected cells and interfering with the trafficking of RuV glycoproteins, E1 and E2 [13]. Unfortunately, NTZ showed limited to no effect on the treatment of iVDRV-associated granulomas in patients, even after long-term treatments [13,16]. The reasons for the failures of NTZ therapy in granuloma treatments are currently not understood. 

Here, we report an investigation of NTZ treatment failures in two cases of RuV-associated granulomas [16]. We tested a hypothesis that continued antiviral treatment with NTZ in patients, with continuing viral replication, can result in the development of drug resistance in iVDRVs. Granuloma samples from two patients with ataxia-telangiectasia (AT) were analyzed, before and after NTZ therapy, for the presence of iVDRV RNA and infectious iVDRVs in granulomatous skin lesions. We also report the sensitivity of iVDRVs to NTZ for the durations of the treatments and the iVDRV quasispecies’ diversity in skin lesions and infectious iVDRV isolates after long-term NTZ therapy.

## 2. Results

### 2.1. Quantitation of iVDRV RNA and Recovery of Infectious iVDRVs from Post-Treatment Biopsies

**Figure 1:**
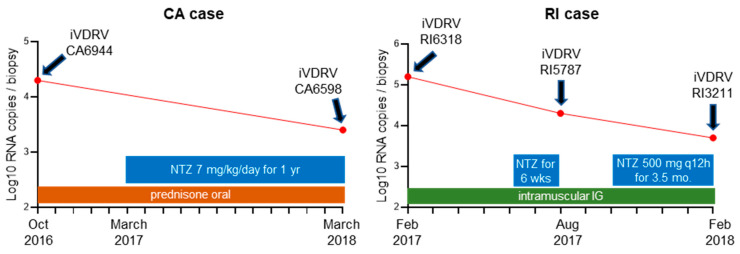
Course of skin granuloma treatments showing the duration of each treatment and oral NTZ dosage. Amounts of iVDRV RNA (the red dots) were determined by RuV RT-qPCR absolute quantification and presented as genomic RNA copies per an entire biopsy. The designations of iVDRV strains isolated from skin lesion biopsies before and at different times after NTZ therapy are shown above the red dots.

Two AT patients (CA and RI) have been described earlier [12,16]. Both patients received MMR vaccination prior to being diagnosed with AT and later developed skin granulomatous lesions, from which infectious iVDRVs (CA6944 and RI6318) were recovered and their whole genomes sequenced [12]. The patients were treated with NTZ as a salvage therapy for iVDRV, without any obvious benefits in either case [16]. NTZ treatment of the CA patient was initiated 5 months after obtaining the T0 skin sample from a lesion on the left arm (Figure 1). The T1 skin biopsy was collected after one-year NTZ treatment from a lesion on the patient’s right arm. NTZ treatment of the RI patient was also initiated 5 months after obtaining the T0 skin sample. The T1 skin biopsy was collected after six weeks of NTZ treatment, which was then suspended for 2.5 months, before being resumed for an additional 3.5 months prior to the collection of the T2 skin sample. All skin biopsies of the RI patient were obtained from the lesion on the patient’s right index finger. We tested T1 and T2 post-treatment samples for the presence of iVDRV RNA and infectious viruses. For both patients, real-time RT-qPCR absolute quantitation analysis revealed reduced amounts of RuV RNA in skin biopsies after NTZ treatments, compared to T0 biopsies (Figure 1). However, infectious iVDRVs were recovered from the T1 and T2 biopsy samples, despite the reduced viral RNA amounts in the tissues. Low passage stocks (passage 4) were prepared for the subsequent experiments. All iVDRV isolates produced similar low virus titers, ranging from 1 × 10^4^ to 3 × 10^4^ foci forming units/ml of cell culture media, suggesting that the iVDRV growth properties did not change after NTZ therapies.

### 2.2. Effects of Tizoxanide on Replication of iVDRV Strains

**Figure 2:**
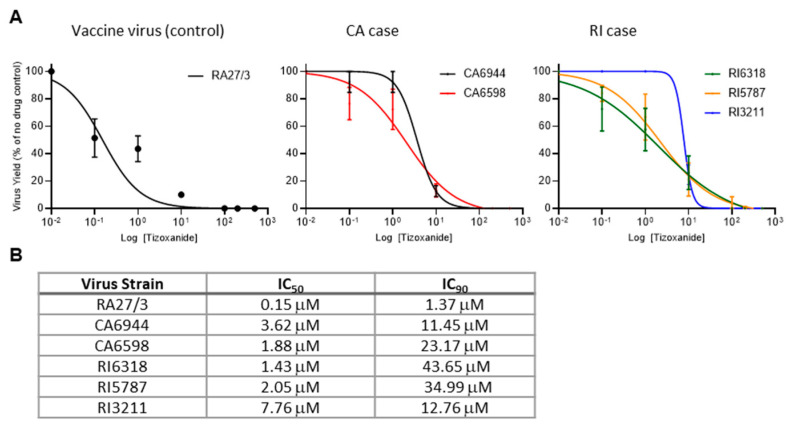
Inhibition of replication of iVDRV strains isolated from skin lesions before and after the NTZ therapies. (**A**). Dose response analysis of A549 cells treated with tizoxanide and infected with iVDRV strains. Data represent the mean of three (RA27/3, CA strains) or four (RI strains) independent experiments +/− SD. (**B**). Table of IC_50_ and IC_90_ of tizoxanide in A459 cells infected with iVDRV strains.

To test whether replication of iVDRVs was inhibited by NTZ, the isolates recovered from the untreated patients (CA6944 and RI6318) were tested for sensitivity to tizoxanide, an active metabolite of NTZ. To determine whether long-term NTZ treatments resulted in the development of drug resistance, tizoxanide sensitivity of the isolates recovered from the lesions after treatment (CA6598, RI5787, and RI3211) were tested and compared to those samples recovered prior to treatment (CA6944 and RI6318). A549 cells were infected with each of the five iVDRV strains and RA27/3 (positive control), at a multiplicity of infection (MOI) of 0.1, treated with varying concentrations of tizoxanide, and then viral yields were determined 48 hours post infection. The iVDRV production in A459 cells was inhibited by tizoxanide in a dose-dependent manner (Figure 2A), but the IC_50_ and IC_90_ values of tizoxanide for iVDRVs were increased by more than 10 times, when compared to those for RA27/3 (Figure 2B). The IC_50_ and IC_90_ values of iVDRV strains were comparable to each other, indicating that no additional resistance to NTZ was developed by the long-term NTZ treatments.

### 2.3. Sequence Analysis of the iVDRV Genomes 

**Figure 3:**
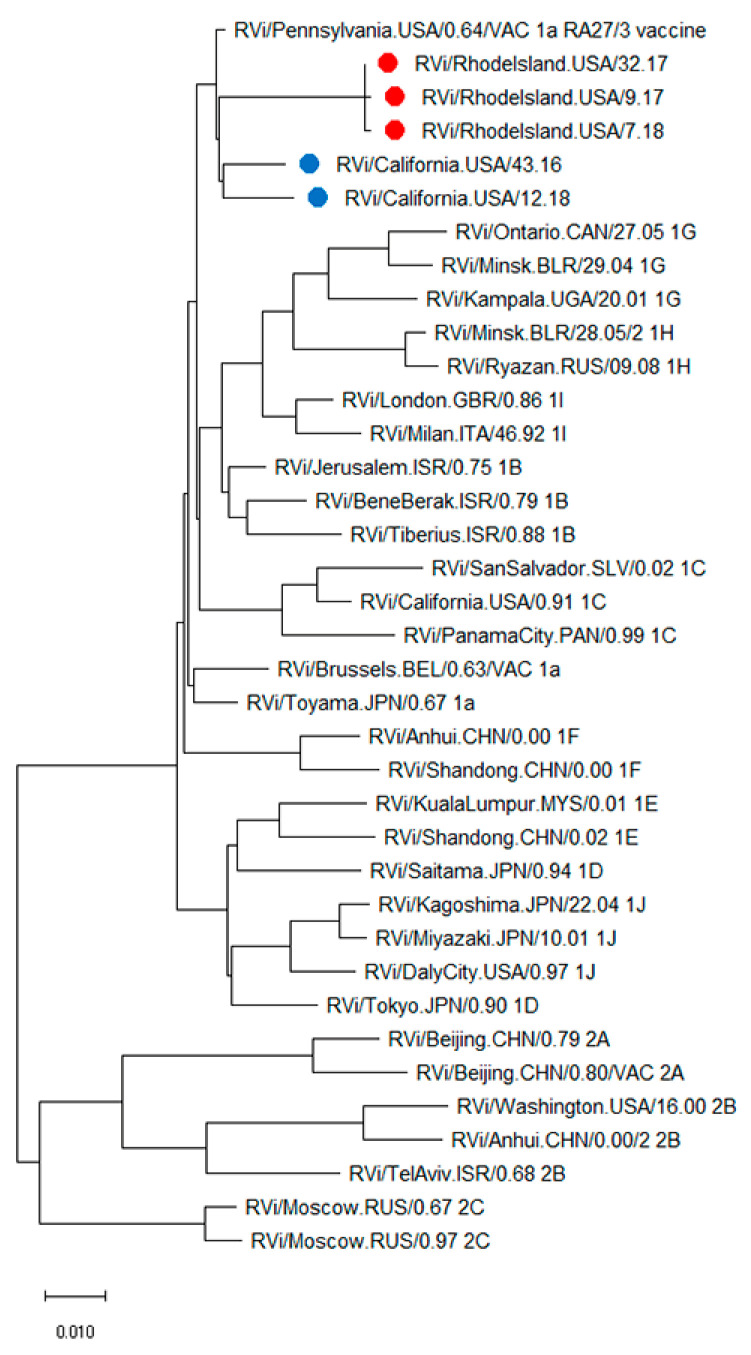
Phylogenetic tree of iVDRV strains. The genetic relationships between the whole genome sequences of iVDRV strains, RA27/3 and the 32 WHO reference viruses were inferred using the Maximum Likelihood method in MEGA10. All taxa are labeled with WHO names with the RI iVDRV sequences marked with red dots and the CA iVDRV sequences marked with blue dots. The scale bar indicates the number of base substitutions per site. RA27/3 and iVDRVs represent a separate branch on the tree with RA27/3 being basal.

Whole genome sequences were obtained from passage 4 viral stocks of CA6598, RI5787, and RI3211, recovered from lesions after NTZ treatments. Phylogenetic analysis of the genome sequences of CA6944, CA6598, RI5787, RI6318, and RI3211, with full genomes of the WHO RuV reference viruses, showed that all patients’ isolates were RA27/3-derived (Figure 3).

**Figure 4:**
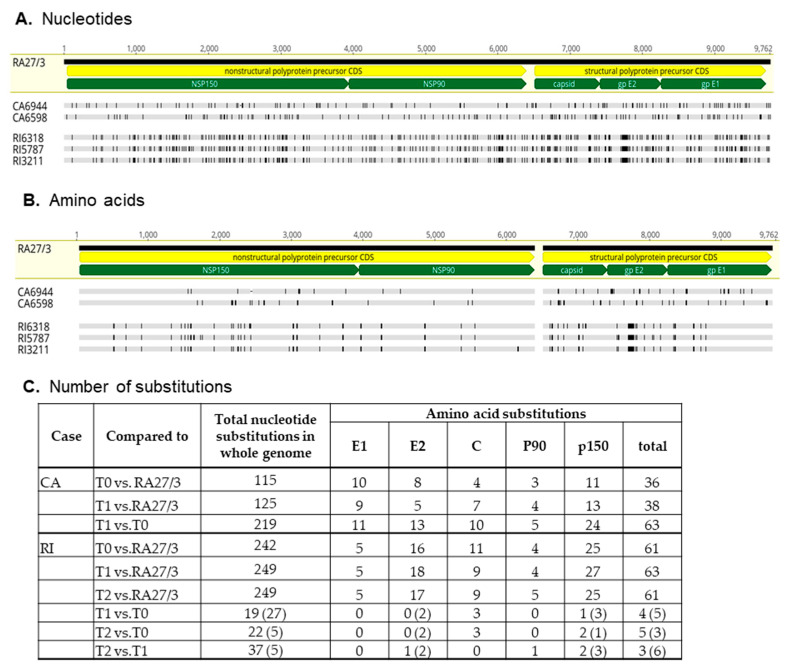
Diversity of iVDRV consensus sequences. (**A**). Nucleotide and (**B**). amino acid substitutions in iVDRV genomes relative to parental RA27/3 virus (GenBank #FJ211588) are depicted as vertical lines. The positions of the coding sequences (CDS) of the nonstructural and structural precursors (yellow pointed bars) and mature protein CDSs (green pointed bars) in the genomic sequence are indicated below the RA27/3 reference sequence. The total number of substitutions are shown for each iVDRV in the table (**C**). The numbers of ambiguous disagreements are in parentheses.

The CA6598, RI5787, and RI3211 genomic sequences were compared to both the RA27/3 parental virus and to the genomic sequences obtained prior to the treatments (CA6944 and RI6318) [12]. The number of nucleotide and amino acid substitutions were almost two times higher between the consensus sequences of CA6944 and CA6598 strains than between each of the CA iVDRV strain and RA27/3 (Figure 4). Most substitutions were unique for each CA iVDRV strain. These data show that CA iVDRVs evolved independently from each other, while persisting in granulomas located on different arms of the same CA patient. As a result, they represent different co-existing viral populations. 

The genomic sequences of the three RI iVDRVs strains were substantially different from the RA27/3 sequence, yet similar to each other, with many nucleotide and amino acid substitutions classified as ambiguous (Figure 4). The most frequent ambiguous disagreement between the RI iVDRV consensus sequences was substitution of C to a C/T mixture of nucleotides, leading to either no amino acid substitutions or no more than three unambiguous amino acid substitutions in RI iVDRV proteins (Figure 4C). These data show that RI iVDRVs were sampled from the same viral population, persisting in the skin lesion on the patient’s finger. The dS and dS nucleotide substitution rates for RI iVDRV were 5.1 × 10^−4^ and 1.7 × 10^−3^ substitutions/site/year, respectively, which are consistent with the iVDRV nucleotide substitution rates reported previously [12]. There were no similar amino acid substitutions in the consensus sequences between iVDRV proteins of CA and RI patients after NTZ treatments. Collectively, these data suggest that NTZ did not exert a specific selective pressure on iVDRV evolution in skin lesions. 

### 2.4. Quasispecies Variability in Skin Lesions and iVDRV Low Passage Isolates

**Figure 5:**
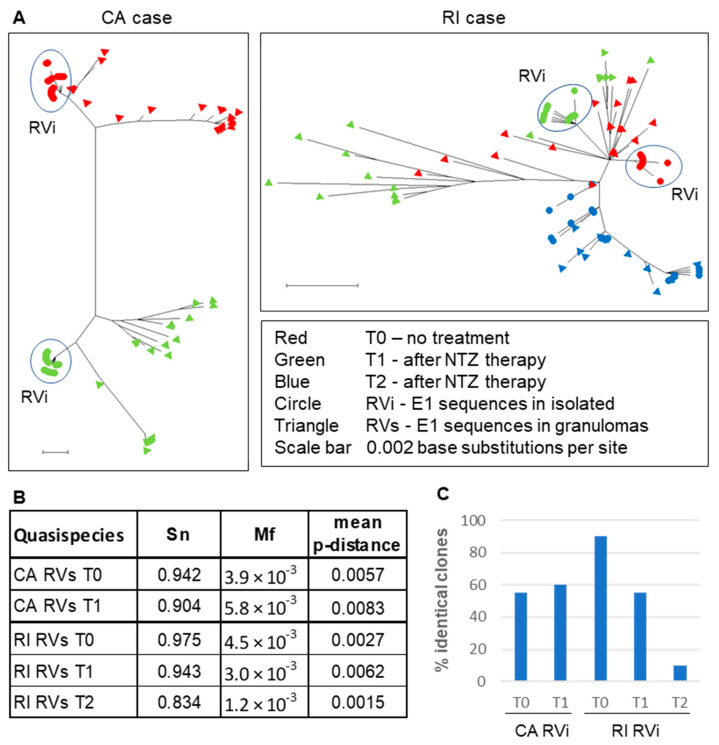
iVDRV quasispecies in tissues and viral isolates before and after NTZ therapy. (**A**). Neighbor-joining tree (non-rooted) for quasispecies within the granuloma samples (triangles) and virus isolates (circles) from the CA and RI cases. The genetic distances were computed using the Maximum Composite Likelihood method and phylogenetic trees were constructed using Mega10 software. (**B**). Diversity parameters of quasispecies in skin lesions. (**C**). Quasispecies diversity in virus isolates.

Our previous study provided evidence that iVDRVs exist not as a single virus species, but rather as a collection of genetically closely related viruses, commonly known as a quasispecies, but also referred to as a mutant spectrum, or mutant swarm [12]. A viral quasispecies acts as a unit of selection and viral microevolution, in response to environmental changes (e.g., drug treatments). Viral sequence evolution often occurs via modification of the mutant spectrum and, therefore, cannot be properly understood from a consensus sequence alone [17]. We investigated whether iVDRV quasispecies diversity in patients’ skin lesions had changed after NTZ therapy. Since E1 intracellular trafficking was shown to be affected by NTZ [13], the E1 gene, a likely NTZ target, was selected for quasispecies diversity analysis by molecular cloning and sequencing approach. The complete E1 gene was amplified from total RNA, isolated from skin lesions and from iVDRV isolates, and then cloned and sequenced. We sequenced 20 E1 clones from each skin sample (RVs E1 clones) and each of the passage 4 iVDRV stocks (RVi E1 clones) (Data S1 and S2). Phylogenetic analysis of the RVi and RVs CA clones revealed non-overlapping mutant clouds in the skin lesions on the left and right patient’s arms, further confirming independent evolution and the coexistence of distinct iVDRV lineages in different body sites of the same patient (Figure 5A). In contrast, the mutant clouds in the skin samples of the RI patient were overlapping, confirming that the same viral population was sampled before and after each of two NTZ treatments. There were no substantial differences in complexity (Sn), diversity (p-distance), or heterogeneity (Mf) parameters, between iVDRV RVs quasispecies in lesions collected before and after treatments of both patients (Figure 5B, Data S3). 

The RVi mutant clouds were less diverse than RVs mutant clouds in T0 and T1 skin samples of both patients and were grouped together on the phylogenetic trees as distinct subpopulations. Nearly half of the CA RVi clones were identical in T0 and T1 skin samples, suggesting RVi quasispecies diversity did not change after NTZ treatment of the CA patient (Figure 5C). The percentage of identical RI RVi clones dropped from 90% (T0) to 10% (T2), showing the substantial increase in quasispecies diversity of replication-competent iVDRVs, after two courses of NTZ in RI patient. None of the RVi clone sequences were identical to the RVs clone sequences, in each of the four corresponding skin samples (CA T0 and T1, RI T0 and T1), except that one out of twenty clones was shared by the RVs and RVi mutant clouds in the RI T2 sample, suggesting that replication-competent iVDRV isolates represent 5% or less of the total viral swarm in the lesions. 

## 3. Discussion

Nitazoxanide treatment of two AT patients with cutaneous granulomas did not provide any benefit or a resolution of rubella-associated granulomatous lesions. In this report, we demonstrated that failure to achieve granuloma resolution was associated with continuous presence of both iVDRV RNA, albeit in reduced quantities, and infectious iVDRVs in lesions, despite the long-term NTZ treatments. Similarly, NTZ substantially reduced viral loads of SARS-CoV-5, influenza viruses, hepatitis C virus and other viruses in patients receiving the drug, compared to patients who received placebo in double-blind, placebo-controlled clinical trials (reviewed in [18,19]). However, despite sustained virological responses, NTZ treatments have not always resulted in symptom resolution, e.g., no accelerated resolution of clinical symptoms was observed in mild COVID-19 disease [20]. 

Initially, the sensitivity of RuV replication to NTZ was investigated using RA27/3 vaccine and wild-type RuV strains, because iVDRV strains were not available at that time [13]. Since NTZ is not a direct antiviral drug, it was predicted that iVDRVs will be as sensitive to NTZ as the parental RA27/3 strain. Unexpectedly, all five iVDRVs recovered from the lesions of both patients, either untreated or NTZ-treated, were 10–40 times less sensitive to tizoxanide than the vaccine strain, with IC_50_ ranging from 1.4 µM to 7.7 µM and IC_90_ ranging from 11.5 µM to 43.7 µM (Figure 2). Plasma concentrations of tizoxanide were shown to vary between 10 µM within 1 h of receiving a standard single dose of 500 mg, to a maximum plasma concentration around 35 µM [21]. It is likely that tizoxanide concentrations in tissues may be lower than in plasma, especially in the middle of dense granuloma formations, where iVDRV-infected cells are located. Similarly, the failure of some drugs to treat Mycobacteria-induced granulomas was associated with poor drug penetration into granulomas [22,23]. Although no efforts were made to determine NTZ penetration, insufficient tizoxanide concentration inside granulomas may be one of the reasons why NTZ therapy failed to clear iVDRV-associated lesions.

The molecular basis of the striking differences in NTZ sensitivity, between the vaccine and vaccine-derived strains, is presently unknown. Possible mechanisms for NTZ antiviral properties were suggested, including enhancements of INF-mediated antiviral responses, inhibition of pro-inflammatory cytokine production, interference with Ca2+ regulatory pathways and ER-to-Golgi trafficking of glycoproteins [19,24,25]. Nevertheless, the precise mechanisms of NTZ antiviral effects are not well understood, making it difficult to explain why NTZ therapies failed in the cases presented here. 

Initially, we suspected the emergence of NTZ resistance as a main cause of therapy failure. Unfortunately, due to the nature of chronic persistent infections, drug resistance may develop rather quickly [26]. However, we did not see any substantial changes in tizoxanide sensitivity in the iVDRV isolates recovered post treatment. Similarly, no antiviral resistance against NTZ was reported among patients treated for influenza viruses [27]. Thus, the emergence of NTZ resistance, following long-term antiviral treatments, appears not to be a major concern, most likely because host-targeted antivirals have low potential for developing drug resistance [28]. 

Understanding quasispecies dynamics is important because of the role that quasispecies play in the potential development of drug resistance. Sequence variations occur randomly throughout the entire viral genomes, but long-term drug treatments during persistent viral replication often result in altered quasispecies distribution of drug target genes, due to the accumulation of resistance mutations [17,29]. We did not observe a reduction in the quasispecies diversity of the E1 gene, a potential NTZ target, in iVDRVs in the skin lesions of either patient after NTZ treatments. E1 quasispecies diversity was also similar between replication-competent iVDRVs recovered from CA patient’s lesions, pre- and post-treatment. In contrast, although the consensus E1 nucleotide and protein sequences did not change after NTZ treatment, E1 quasispecies diversity of replication-competent RI iVDRVs increased substantially, from 90% identical clones in the untreated patient to 10% identical clones after two NTZ courses. High diversity in the RI3211 iVDRV viral stock may explain the differences in slope of the dose response curve for this iVDRV, compared to the curves for the other two iVDRVs of this patient (Figure 2). Since an exact antiviral mechanism of NTZ is presently unknown, the diversification of RI iVDRV E1 quasispecies in the T2 sample is likely due to genetic drift, rather than NTZ selective pressure. It should be noted that iVDRV RNA load in the T2 sample was reduced by more than one log, compared to the T0 sample, suggesting a population bottleneck effect. Studies of viral sequence evolution in chronically infected hosts provided strong evidence that quasispecies diversification plays an important role in evading control by host immunity and drug interventions, resulting in virus persistence [17,29].

This is the second report that provides strong evidence of independent iVDRV evolution in lesions located in different anatomic sites. Previously, we have identified distinct iVDRV lineages in a skin lesion and nasopharynx of the same patient [12]. Herein, coexistence of different iVDRVs lineages on both arms of the CA patient were supported by the number of substitutions between iVDRVs from different arms being substantially higher than the number of substitutions between each iVDRV and RA27/3 genomes, the lack of overlapping mutations in iVDRVs, and non-overlapping iVDRV mutant clouds in both lesions. Such compartmentalization of viral quasispecies and their independent evolution can lead to amplified diversification of persisting iVDRVs in an individual and enhancement of iVDRV resistance to immune control and drug interventions. Ultimately, quasispecies diversity has the long-term potential to affect viral pathogenesis and viral evolution [30,31].

Although sweeping conclusions cannot be made due to the low number of patients, this report provides important observations about possible causes of the failure of NTZ therapy to treat rubella-associated granulomas. This report provides the first evidence of the substantially reduced sensitivity of iVDRVs to tizoxanide, compared to the RA27/3 vaccine strain. This report also documents the lack of NTZ resistance emergence over time, as well as describing NTZ effects on the iVDRV quasispecies diversity. The development of direct RuV-specific antiviral drugs is warranted to efficiently eliminate the persisting virus in rubella-associated granulomas in the vulnerable population of patients, mostly children, with inborn errors of immunity.

## 4. Materials and Methods

### 4.1. Patients Samples

Skin biopsy samples of CA patient were collected from the granuloma on the upper-left arm prior to NTZ treatment (T0) and from the granuloma on the upper-right arm after one-year-treatment (T1). Skin biopsy samples of RI patient were collected from granulomas on the right index finger (RI case) prior to NTZ treatment (T0), and after six weeks (T1) and six months of NTZ treatment (T2) (Figure 1). The biopsies were immediately frozen on dry ice and sent to the Centers for Disease Control and Prevention (CDC, Atlanta, GA, USA) for molecular testing and cell culture. Please note that designations “CA” and “RI” refer only to the state where the patient was seen and do not contain any patient identifying information. 

### 4.2. Cell and Virus Cultures

Vero cells (ATCC #CCL81) and A549 cells (ATCC #CCL185) were cultured in Dulbecco’s Modified Eagle Medium (Thermo Fisher Scientific, Waltham, MA, USA), supplemented with 5% (Vero) or 10% (A549) fetal bovine serum (FBS, Atlanta Biologicals, Flowery Branch, GA, USA) and 50 μg/mL gentamycin (Thermo Fisher Scientific, Waltham, MA, USA). 

Infectious iVDRV isolates were recovered from snap-frozen skin biopsy tissues. Skin tissues were homogenized in DMEM media supplemented with 2% FBS and 1% antibiotic/antimycotic solution (Thermo Fisher Scientific, Waltham, MA, USA) using 3-mm zirconium beads (Sigma-Aldrich, Burlington, MA, USA) and then inoculated into subconfluent monolayers of Vero cells as previously described [12]. Passage 2 iVDRV viral stocks were used as “golden stocks” for subsequent propagation. Preparation of concentrated iVDRV viral stocks (passage 4) with a Jumbosep 300K centrifugal device (PALL Life Sciences, New York, NY, USA), and determination of viral titers by immunocolorimetric foci assay have been published previously [12]. RuV presence in the culture medium was confirmed by real-time RT-PCR using a QuantiFast Multiplex RT-PCR Kit (Qiagen, Hilden, Germany) and rubella virus-specific primers and probes [12]. 

### 4.3. Drug Sensitivity and Viral Response Analyses

A549 cells plated in 96-well plates at a density of 2 × 10^4^ per well were infected at an MOI of 0.1 with RuV strains and then treated with tizoxanide (Cayman Chemical Company, Ann Arbor, MI, USA) at various concentrations. The medium from each well was collected at 48 hours after infection and viruses were tittered on Vero cells [12]. Log (inhibitor) versus RuV yield-variable slope analysis was done using GraphPad prism 7 to determine IC_50_ and IC_90_ values. RuV yields were expressed as a percentage of untreated control. 

### 4.4. Molecular Analyses

RuV RNA was isolated from frozen biopsies using the RNeasy Fibrous Tissue Mini Kit (Qiagen) according to the kit instructions. Quantitative rubella real-time RT-qPCR has been described elsewhere [23]. iVDRV RNA was isolated from viral stocks using the Viral RNA Mini Kit (Qiagen) and converted into cDNA using SuperScript IV Reverse Transcriptase (Thermo Fisher Scientific, Waltham, MA, USA). Five overlapping genomic PCR fragments were amplified from iVDRV cDNA using the Extensor Hi-Fidelity PCR Master Mix (Thermo Fisher) and sequenced by the Sanger method as described [12]. The iVDRV genomic sequences were alighted, annotated, and the number of nucleotide and amino acid substitutions were determined using Geneious software version 11.1.2 (Biomatters LTD, Auckland, New Zealand). The graphic presentation of iVDRV genomes and the location of nucleotide and amino acid substitutions was prepared with Geneious software. The phylogenetic tree of iVDRV whole genome sequences was constructed using the Maximum Likelihood method and Tamura–Nei model using Mega10 software [32].

### 4.5. Quasispecies Analyses

cDNA was synthesized using the Superscript IV First-Strand Synthesis System (Thermo Fisher Scientific, Waltham, MA, USA) according to the manufacturer’s instructions. A 1.4 kb fragment containing the full rubella E1 gene was amplified using Platinum SuperFi DNA Polymerase (Thermo Fisher Scientific, Waltham, MA, USA) and primers RUB8219F (5′-GCGGTCGTCCTGCAGGGKTA-3′) and RUB9762R (5′-T18CTATACAGCAACAGGTGC-3′). The PCR fragments were cloned into a pCR-Blunt II-TOPO vector using a Zero Blunt PCR Cloning Top10 kit (Thermo Fisher Scientific, Waltham, MA, USA). The recombinant clones were selected on imMedia Kanamycin-supplemented agar plates (Thermo Fisher Scientific, Waltham, MA, USA). Plasmid DNA was purified using a ZymoPURE Plasmid Miniprep kit (Zymo Research, Irvine, CA, USA). The plasmid inserts were sequenced by a Sanger method using an ABI BigDye 3.1 kit and ABI 3500 sequencer (Thermo Fisher Scientific, Waltham, MA, USA). The following primers were used for sequencing: RUB8219F, RUB9762R, RUB9313F (5′-AAGTGCGGRCTCCACATACG-3′), RUB8633F (5′-AGCGACGCGGCCTGCTGGGG-3′), and RUB9112R (5′-GCGCGCCTGAGAGCCTATGAC-3′). Full E1 sequences for each clone were assembled using a SeqMan Pro software (DNAstar program suite, v.15). 

E1 nucleotide and amino acid sequences were aligned using MUSCLE algorithm with Mega10 [32] or Geneious software (Biomatters LTD, v.11.1.2). Three parameters were used to characterize quasispecies complexity in lesions: normalized Shannon entropy (Sn), mutation frequency (Mf), and nucleotide diversity (*p*-distance) [33]. P-distance matrixes and mean p-distance were calculated using Mega10 [32]. Sn and Mf were calculated using the equations suggested by Gregori et al. [33] (Data S3). Phylogenetic trees of quasispecies were constructed using the Maximum Likelihood method and Tamura–Nei model using Mega10 software [32]. The dS and dN substitution rates were calculated as previously described [12].

## Data Availability

Complete sequences of iVDRV genomes have been deposited in the NCBI database under accession numbers MK787188, MK787189, and OM022830- OM022832.

## Supplementary Materials

The following supporting information can be downloaded at: https://www.mdpi.com/article/10.3390/pathogens11030338/s1, Data S1: RVi E1 clones FastA; Data S2: RVs E1 clones FastA; Data S3: Quasispecies Diversities.
