# Supplementary material for: Drug Sensitivity of Vaccine-Derived Rubella Viruses and Quasispecies Evolution in Granulomatous Lesions of Two Ataxia-Telangiectasia Patients Treated with Nitazoxanide
Source: Pathogens. 2022 Mar 11;11(3):338. doi: 10.3390/pathogens11030338 (PMC8955873; doi:10.3390/pathogens11030338)
Supplement: Supplementary file 1 [file pathogens-11-00338-s001.zip › msQS-NTZ Figs v1.pptx]

## Slide 1
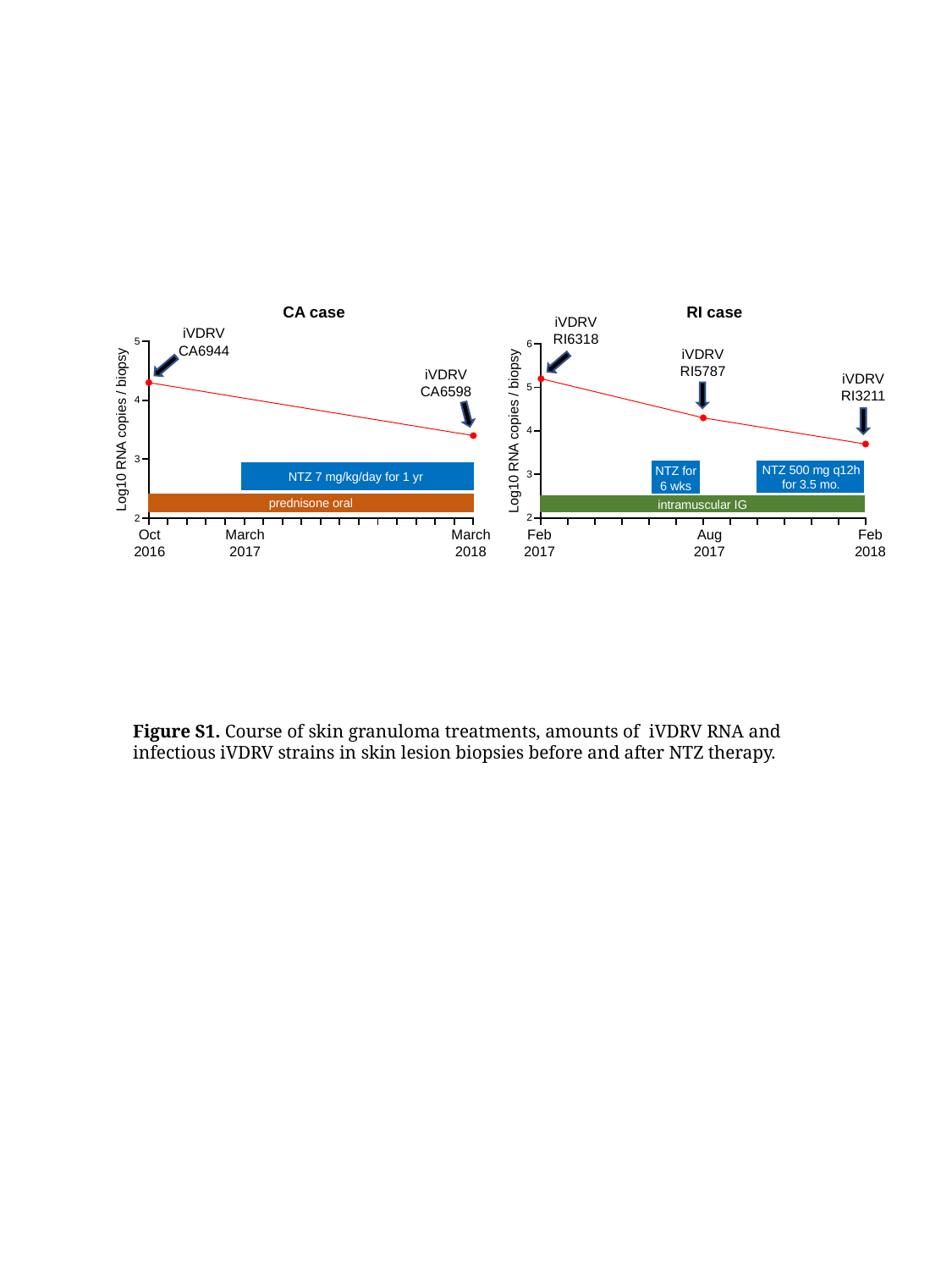

CA case
RI case
iVDRV RI6318
iVDRV CA6944
iVDRV RI5787
iVDRV CA6598
iVDRV RI3211
NTZ 500 mg q12h for 3.5 mo.
NTZ for 6 wks
NTZ 7 mg/kg/day for 1 yr
prednisone oral
intramuscular IG
Oct 2016
March 2017
March 2018
Feb 2017
Aug
2017
Feb 2018
Figure S1. Course of skin granuloma treatments, amounts of iVDRV RNA and infectious iVDRV strains in skin lesion biopsies before and after NTZ therapy.

## Slide 2
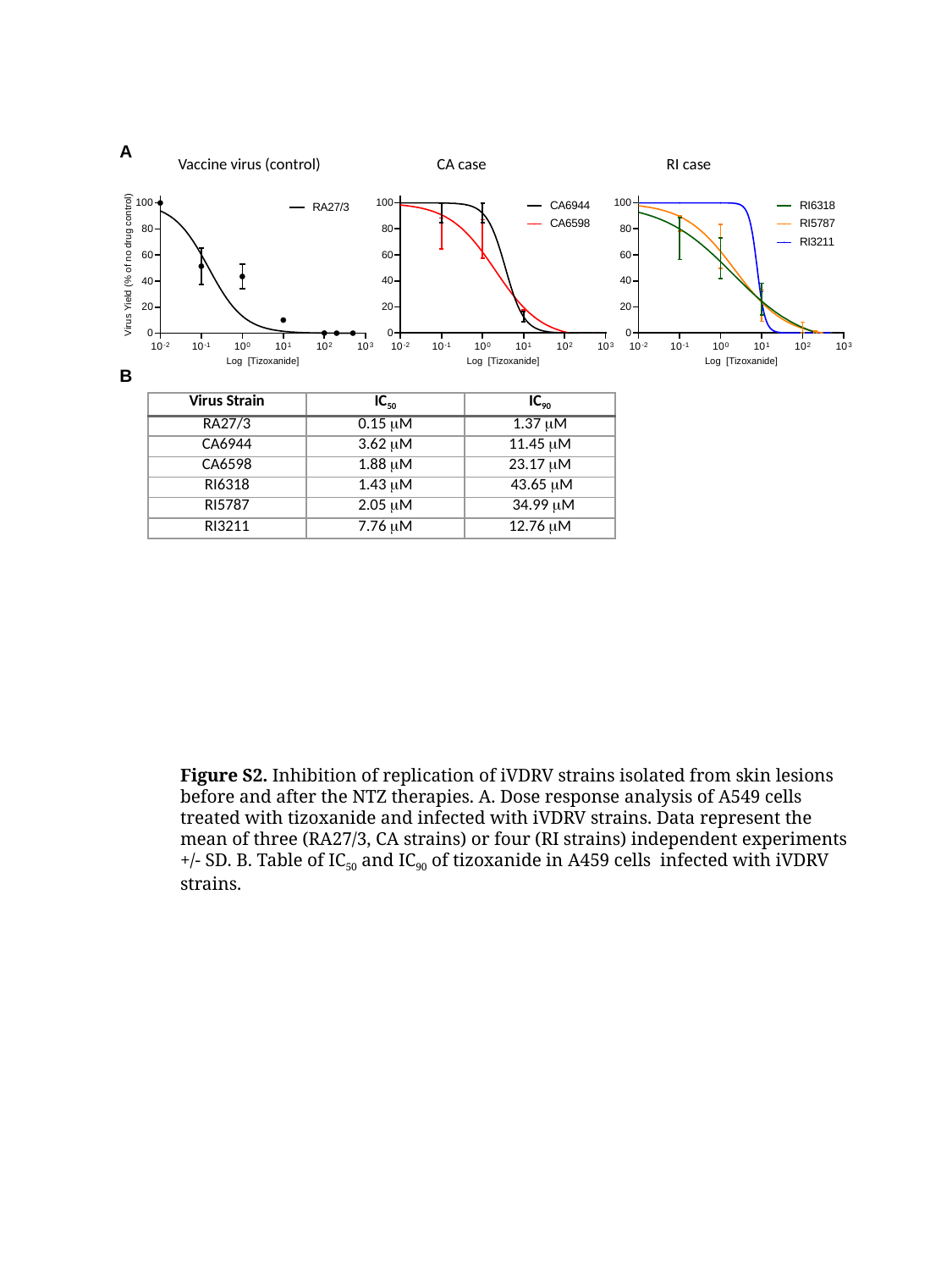

A
Vaccine virus (control)
CA case
RI case
B
| Virus Strain | IC50 | IC90 |
| --- | --- | --- |
| RA27/3 | 0.15 mM | 1.37 mM |
| CA6944 | 3.62 mM | 11.45 mM |
| CA6598 | 1.88 mM | 23.17 mM |
| RI6318 | 1.43 mM | 43.65 mM |
| RI5787 | 2.05 mM | 34.99 mM |
| RI3211 | 7.76 mM | 12.76 mM |
Figure S2. Inhibition of replication of iVDRV strains isolated from skin lesions before and after the NTZ therapies. A. Dose response analysis of A549 cells treated with tizoxanide and infected with iVDRV strains. Data represent the mean of three (RA27/3, CA strains) or four (RI strains) independent experiments +/- SD. B. Table of IC50 and IC90 of tizoxanide in A459 cells infected with iVDRV strains.

## Slide 3
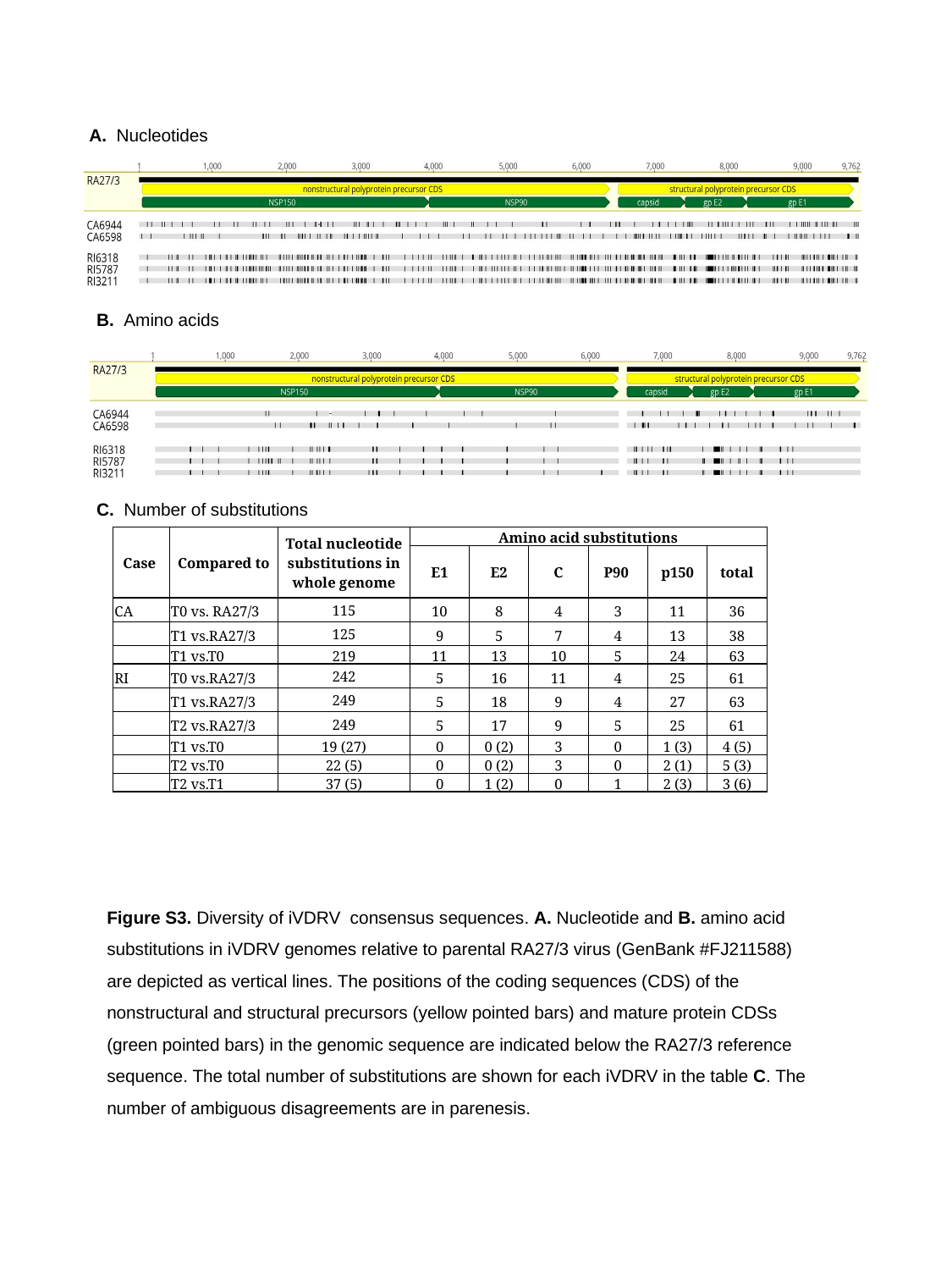

A. Nucleotides
B. Amino acids
C. Number of substitutions
| Case | Compared to | Total nucleotide substitutions in whole genome | Amino acid substitutions | | | | | |
| --- | --- | --- | --- | --- | --- | --- | --- | --- |
| | | | E1 | E2 | C | P90 | p150 | total |
| CA | T0 vs. RA27/3 | 115 | 10 | 8 | 4 | 3 | 11 | 36 |
| | T1 vs.RA27/3 | 125 | 9 | 5 | 7 | 4 | 13 | 38 |
| | T1 vs.T0 | 219 | 11 | 13 | 10 | 5 | 24 | 63 |
| RI | T0 vs.RA27/3 | 242 | 5 | 16 | 11 | 4 | 25 | 61 |
| | T1 vs.RA27/3 | 249 | 5 | 18 | 9 | 4 | 27 | 63 |
| | T2 vs.RA27/3 | 249 | 5 | 17 | 9 | 5 | 25 | 61 |
| | T1 vs.T0 | 19 (27) | 0 | 0 (2) | 3 | 0 | 1 (3) | 4 (5) |
| | T2 vs.T0 | 22 (5) | 0 | 0 (2) | 3 | 0 | 2 (1) | 5 (3) |
| | T2 vs.T1 | 37 (5) | 0 | 1 (2) | 0 | 1 | 2 (3) | 3 (6) |
Figure S3. Diversity of iVDRV consensus sequences. A. Nucleotide and B. amino acid substitutions in iVDRV genomes relative to parental RA27/3 virus (GenBank #FJ211588) are depicted as vertical lines. The positions of the coding sequences (CDS) of the nonstructural and structural precursors (yellow pointed bars) and mature protein CDSs (green pointed bars) in the genomic sequence are indicated below the RA27/3 reference sequence. The total number of substitutions are shown for each iVDRV in the table C. The number of ambiguous disagreements are in parenesis.

## Slide 4
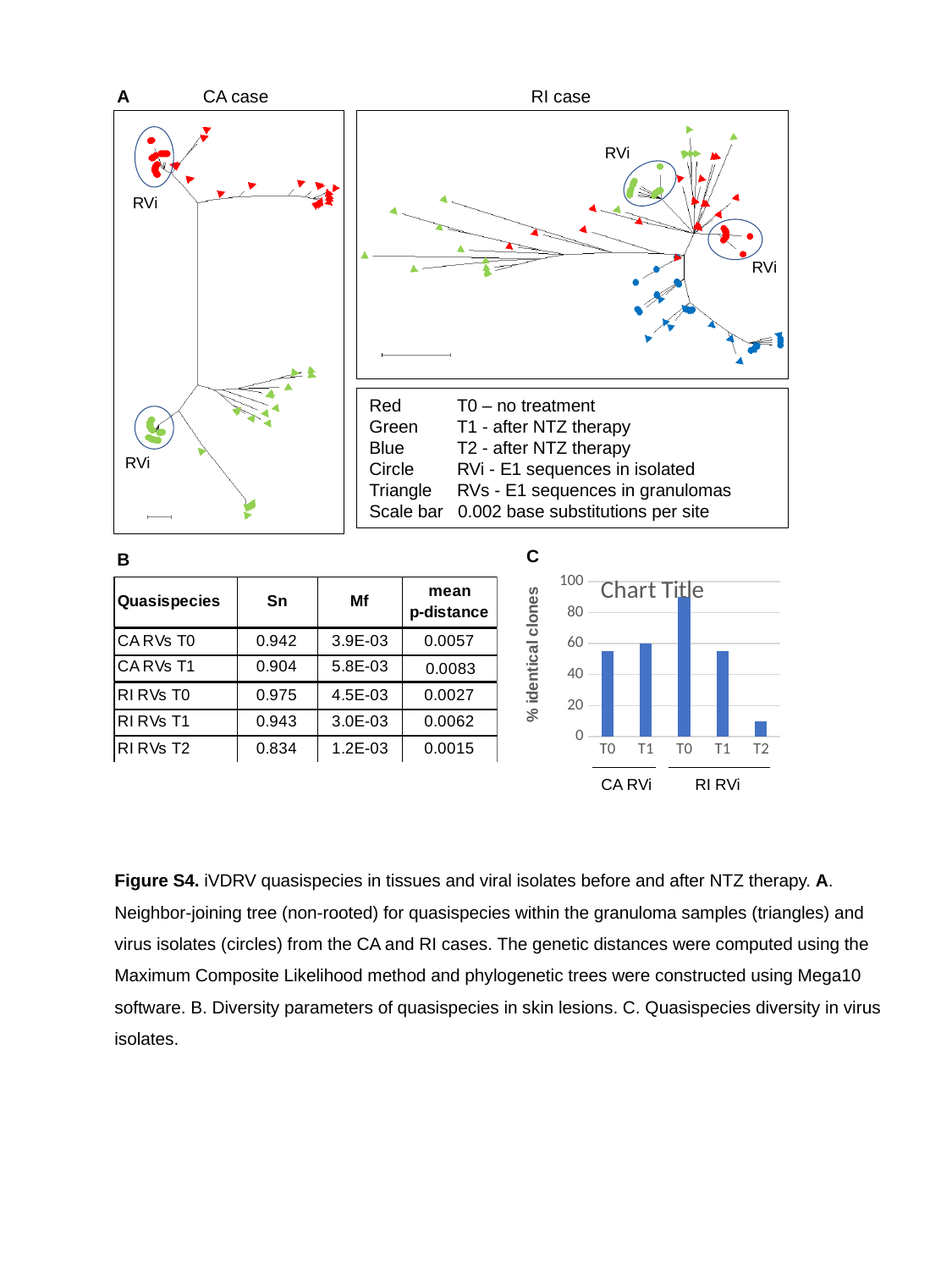

A
RI case
CA case
RVi
RVi
RVi
Red 	T0 – no treatment
Green 	T1 - after NTZ therapy
Blue 	T2 - after NTZ therapy
Circle 	RVi - E1 sequences in isolated
Triangle 	RVs - E1 sequences in granulomas
Scale bar 0.002 base substitutions per site
RVi
C
B
### Chart:
| Category | |
|---|---|
| T0 | 55.0 |
| T1 | 60.0 |
| T0 | 90.0 |
| T1 | 55.0 |
| T2 | 10.0 |CA RVi
RI RVi
Figure S4. iVDRV quasispecies in tissues and viral isolates before and after NTZ therapy. A. Neighbor-joining tree (non-rooted) for quasispecies within the granuloma samples (triangles) and virus isolates (circles) from the CA and RI cases. The genetic distances were computed using the Maximum Composite Likelihood method and phylogenetic trees were constructed using Mega10 software. B. Diversity parameters of quasispecies in skin lesions. C. Quasispecies diversity in virus isolates.
